# Community-Acquired Klebsiella pneumoniae Meningitis With Liver Abscess and Neurological Sequelae in an Immunocompetent Adult: A Case Report

**DOI:** 10.7759/cureus.116236

**Published:** 2026-09-14

**Authors:** Ghamdan Alsadeh, Sheema T Parakkal, Leyla K Bereka, Ahmed S Rahal

**Affiliations:** 1 Critical Care Medicine, Ibrahim Bin Hamad Obaidullah Hospital, Emirates Health Services (EHS), Ras Al Khaimah, ARE

**Keywords:** acute hemiplegia, community-acquired meningitis, klebsiella pneumoniae (kp), klebsiella pneumoniae meningitis, liver abscess aspiration

## Abstract

*Klebsiella pneumoniae* meningitis (KPM) is a rare but severe form of community-acquired bacterial meningitis that is often associated with high mortality and neurological sequelae. We report a 46-year-old previously healthy male who presented with acute altered mental status and high-grade fever. Cerebrospinal fluid analysis was consistent with bacterial meningitis, and cultures from the cerebrospinal fluid and blood showed *Klebsiella pneumoniae (K. pneumoniae)* growth. The clinical course of the patient was complicated with seizures and acute right-sided hemiplegia. Persistent fever (despite appropriate antimicrobial therapy) prompted further evaluation, which revealed a hepatic abscess. Percutaneous abscess drainage resulted in rapid clinical improvement and notable neurological recovery. This case highlights the importance of early recognition of *K. pneumoniae* as a potential cause of community-acquired meningitis and the need to actively search for an underlying primary source, particularly a liver abscess, when the clinical response to antibiotics is suboptimal.

## Introduction

*Klebsiella pneumoniae (K. pneumoniae)*, a gram-negative bacterial pathogen, is frequently associated with nosocomial infections, including pneumonia, urinary tract infections, and bloodstream infections. In 1980, hypervirulent *Klebsiella pneumoniae* (hvKp) was first identified in Taiwan, resulting in severe community-acquired liver abscesses that predominantly affected healthy individuals. Since its initial identification, hvKp infections, including meningitis, liver abscess, and metastatic dissemination, have been documented throughout Asia and recently in America, Europe, and Australia [1, 2]. Recent epidemiological data from Korea (2009-2023) indicate that *K. pneumoniae* has emerged as the most prevalent etiological agent of adult community-acquired bacterial meningitis, whereas the incidence of *Streptococcus pneumoniae* has declined [3]. Although antibiotics effectively eliminate bacteria from the cerebrospinal fluid, the mortality rate persists at approximately 25%, with 21-28% of survivors suffering from neurological impairment [4]. *K. pneumoniae* is of increasing concern owing to its high treatment resistance and poor prognosis, particularly in meningitis cases [5, 6]. Our case highlights a rare occurrence of *K. pneumoniae*-induced meningitis associated with liver abscess in an immunocompetent patient.

Written informed consent was obtained from the patient for publication of this case report. Patient confidentiality was maintained, and no identifying information is included in the manuscript.

## Case presentation

A 46-year-old male smoker, with no relevant medical history, was brought to the emergency department in November 2025 after developing acute confusion and agitation at his workplace. Upon arrival, the patient was agitated with confused speech, Glasgow Coma Scale (GCS) score of 10 (E3V4M3). Both pupils measured 2 mm bilaterally and were reactive. He was febrile with a temperature of 39.10°C, with sinus tachycardia, and well perfused with blood pressure maintained within the normal range. Cardiovascular and chest auscultation were normal, and abdominal examination, apart from mild tenderness, was unremarkable. Neurological examination revealed neck stiffness and a positive Kernig's sign, with no initial focal neurological deficits. Laboratory investigations showed leukocytosis (17 × 10³/µL) with neutrophilic predominance of 86%, elevated inflammatory markers (C-reactive protein 402 mg/L, procalcitonin 21 µg/L), and lactic acidosis (4.5 mmol/L). The hemoglobin level, platelet count, renal function, liver enzymes, and coagulation profiles were within normal ranges (Table 1). Blood cultures were obtained from two separate sites. The initial non-contrast computed tomography (CT) scans of the brain and cervical spine were unremarkable. A lumbar puncture revealed an elevated opening pressure of 20-25 cmH₂O. The cerebrospinal fluid (CSF) appeared turbid (Figure 1).

**Table 1:** Laboratory results at admission and discharge CRP - C-reactive protein; ICU - intensive care unit; PCT - procalcitonin; WBC - white blood cell count

Laboratory tests	At admission to ICU	At discharge from ICU	Reference range
WBC	17 × 10^3^/mcL	9 × 10^3^/mcL	4-10 × 10^3^/mcL
Neutrophils	86%	75%	40–70%
Hemoglobin	16 gm/dL	14 gm/dL	13-17 gm/dL
Platelets count	199 × 10^3^/mcL	310 × 10^3^/mcL	150-450 × 10^3^/mcL
PCT	21 ug/L	4.39 ug/L	<0.10
CRP	402 mg/L	160 mg/L	0.00-3.00 mg/L

**Figure 1:**
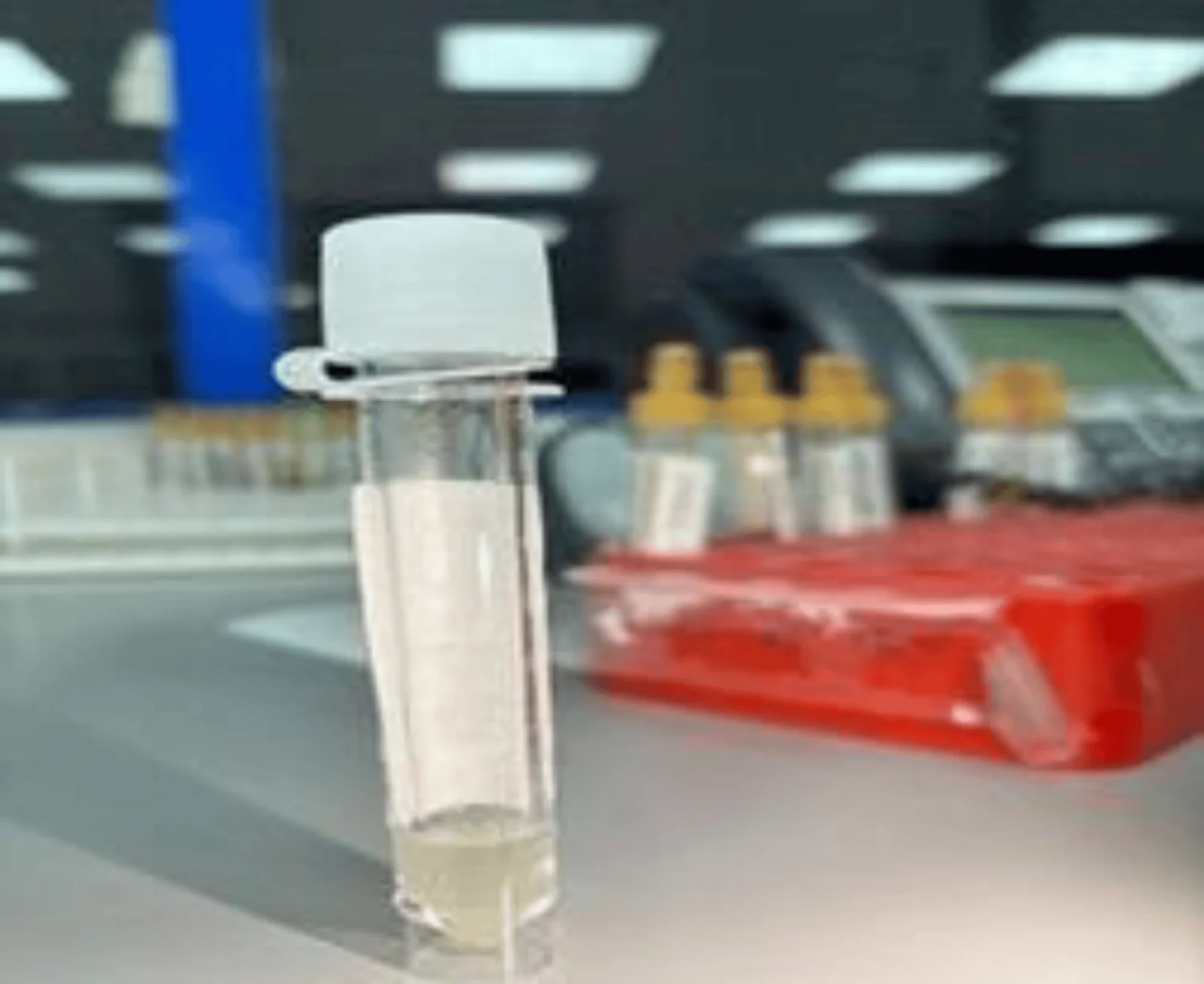
Gross appearance of the cerebrospinal fluid (CSF) sample. The turbid appearance is consistent with bacterial meningitis.

CSF analysis demonstrated a white cell count of 10,778 cells/µL (80% neutrophils), low glucose (0.3 mmol/L, simultaneous serum glucose 13 mmol/L), markedly elevated protein (1082 mg/L), and elevated CSF lactate (25.8 mmol/L). Gram staining, acid-fast bacilli smears, and viral polymerase chain reaction (PCR, HSV-1 and HSV-2) results were negative (Table 2).

**Table 2:** Cerebrospinal fluid (CSF) analysis result AFB - acid-fast bacilli; CSF - cerebrospinal fluid; LDH - lactate dehydrogenase; MN - monocyte; PMN - polymorphonuclear; RBC - red blood cells

Parameter	Results	Reference range
CSF opening pressure (cmH₂O)	20-25 cmH₂O	10-20 cmH₂O
Appearance	turbid	
WBC	10,778 cells/uL	0-5 cells/uL
PMN	80%	0-2%
MN	20%	0-2%
RBC	622 cells/uL	0-5 cells/uL
Protein	1082 mg/L	150-450 mg/L
Glucose	0.3 mmol/L (synchronous blood glucose 13 mmol/L)	2.2-3.9 mmol/L
Lactic Acid	25.8 mmol/L	0.6-2.2 mmol/L
LDH	548 IU/L	<25 IU/L
Gram stain	No organism	
Herpes simplex virus type 1 and type 2 DNA	No organism	
AFB smear	No organism	
CSF culture	Klebsiella pneumoniae isolated	

The patient was admitted to the intensive care unit and was empirically treated with intravenous ceftriaxone 2 g every 12 hours, intravenous vancomycin 1 g every 12 hours, and intravenous dexamethasone 6 mg every six hours. Levetiracetam 500 mg was administered intravenously every 12 hours, with diazepam 5 mg administered intravenously as needed for seizure control. Further laboratory tests for human immunodeficiency virus (HIV), thyroid function, vasculitis screening, *Brucella *IgG, and tuberculosis infection yielded negative results.

On the second day of intensive care unit (ICU) admission, the patient developed generalized tonic-clonic seizures, which were aborted with intravenous diazepam and subsequently managed with levetiracetam. Neurological examination revealed a decline in mental status, a Glasgow Coma Scale (GCS) score of 10 (E3V2M5), and a new-onset right-sided neurological deficit, including facial weakness with mouth deviation to the right side and dense right upper limb monoplegia (0/5). Repeated CT brain imaging and angiography excluded acute ischemic or hemorrhagic strokes. Brain magnetic resonance imaging (MRI) was not performed due to financial constraints.

On day three of ICU admission, the CSF culture grew *K. pneumoniae* (Figure 2A). Two sets of peripheral blood cultures also grew *Klebsiella pneumoniae* (Figure 2B).

**Figure 2:**
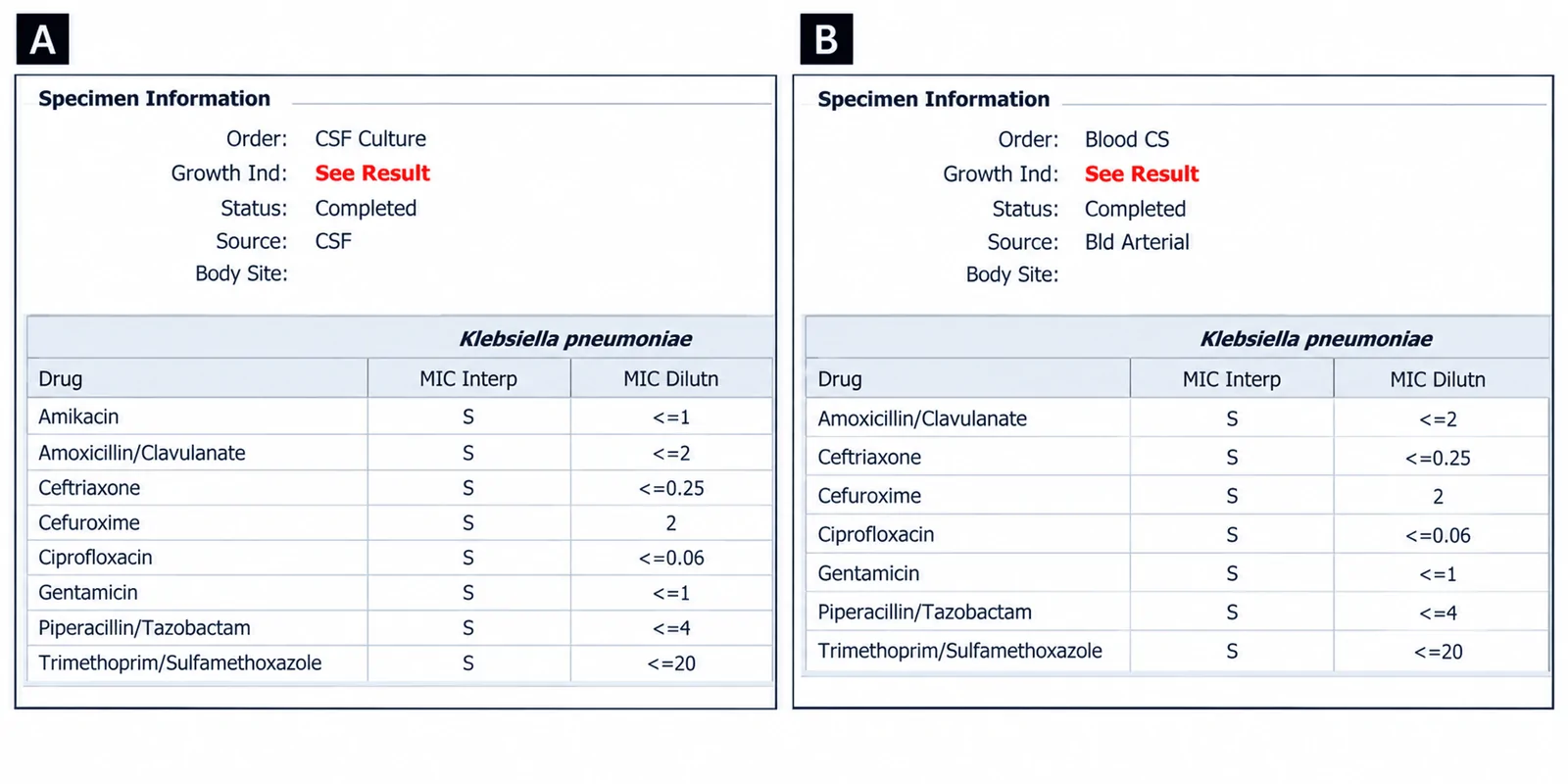
Microbiological findings from cerebrospinal fluid and blood cultures (A) Cerebrospinal fluid culture showing growth of *Klebsiella pneumoniae* with antimicrobial susceptibility results. (B) Blood culture showing growth of *Klebsiella pneumoniae* with antimicrobial susceptibility results.

These findings led to the diagnosis of *Klebsiella *meningitis with a focal neurological deficit. Specialized hypervirulence testing was not performed at our facility due to its unavailability. Based on the disease severity and central nervous system involvement, antimicrobial therapy was escalated to intravenous meropenem 1 g every eight hours, initiated on 17 November 2025, and subsequently increased to 2 g every eight hours from 24 November 2025. Vancomycin 1 g every 12 hours was discontinued on 17 November 2025. Meropenem was continued until 3 December 2025. Despite receiving appropriate therapy, the patient continued to experience high-grade fever and chills. A contrast-enhanced CT scan of the abdomen performed on day five revealed a 6 × 7 cm hypodense lesion with peripheral enhancement in the left hepatic lobe, consistent with a liver abscess, along with a small lesion in segment IVb (Figure 3). Percutaneous drainage of the liver abscess was performed (Figure 4).

**Figure 3:**
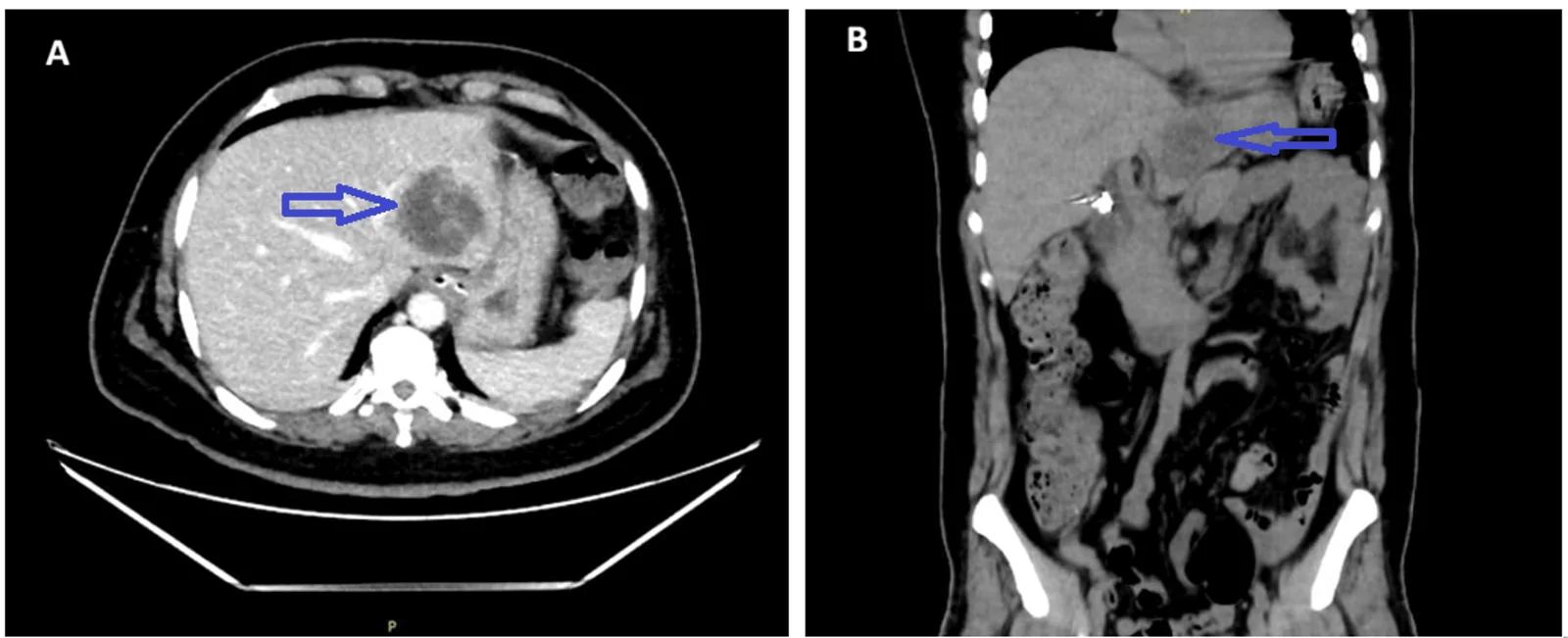
Axial (A) and coronal (B) contrast-enhanced CT images showing a 6 × 7 cm hypodense lesion in the left hepatic lobe with multiple fine enhancing septations and peripheral enhancement (blue arrows), consistent with a liver abscess. An additional small lesion was identified in segment IVb

**Figure 4:**
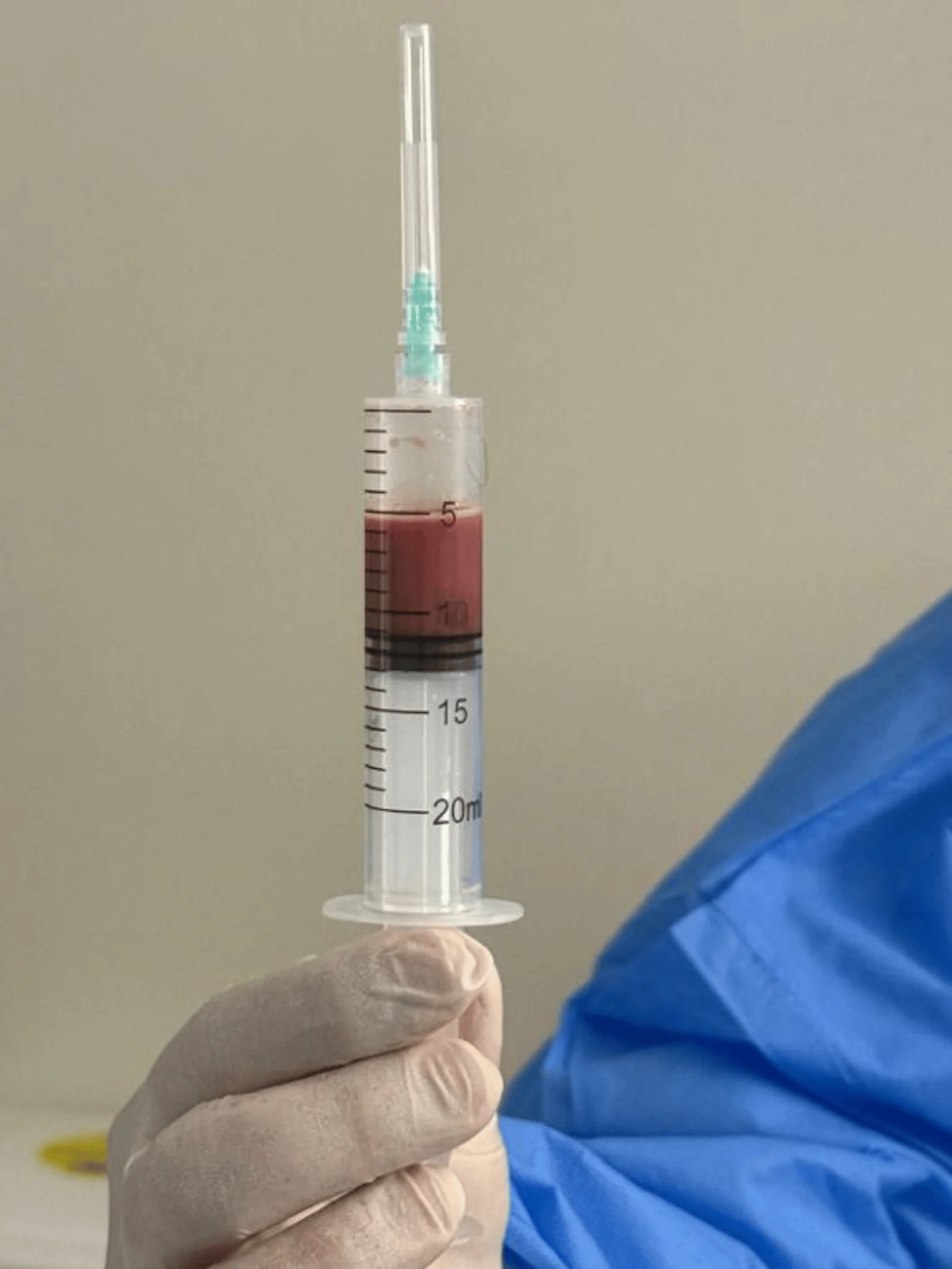
Percutaneous drainage of the liver abscess

Following abscess drainage, the patient showed significant clinical improvement, with resolution of fever and stabilization of overall clinical condition. Neurological recovery was observed, with gradual improvement in right-sided motor strength beginning on day seven of hospitalization. Intensive physiotherapy and early mobilization were initiated. Antibiotics were subsequently de-escalated to ceftriaxone 2 g every 12 hours. The patient completed antibiotic therapy and was transferred to the medical ward in stable condition. He was discharged on 7 December 2025 with near-complete neurological recovery, except for mild residual hearing impairment. Following discharge, the patient returned to his home country. No further clinical follow-up information was available. The timeline of the key clinical events is presented in Figure 5.

**Figure 5:**
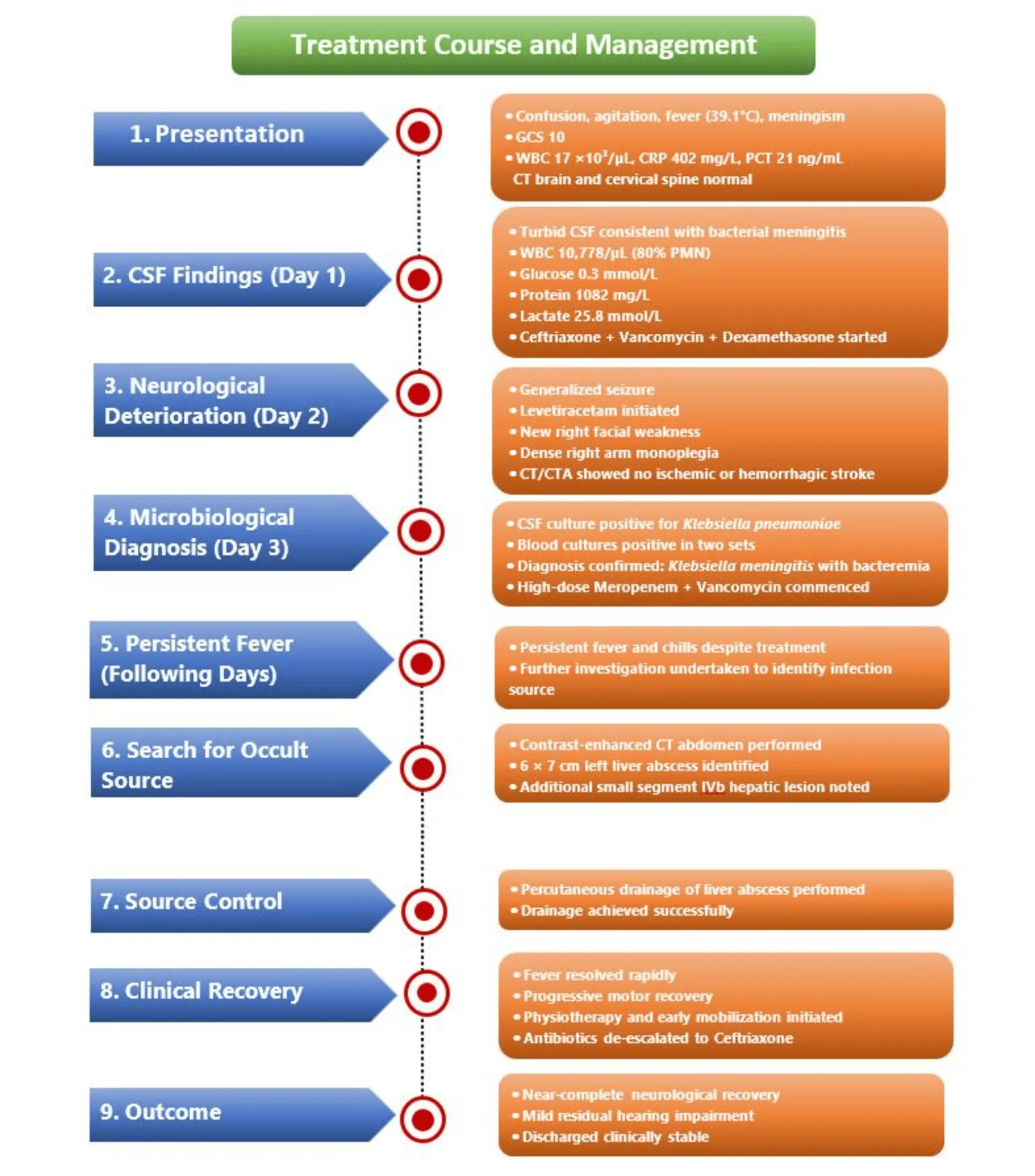
A timeline of the key clinical events and management WBC - white blood cells; CRP - C-reactive protein; PCT - procalcitonin; CSF - cerebrospinal fluid

## Discussion

*K. pneumoniae* meningitis (KPM) is generally infrequent but is associated with high mortality. KPM is considered the third leading cause of meningitis-related deaths worldwide [7]. Various risk factors identified include postoperative individuals or patients with catheters, immunocompromised elderly patients, those with uncontrolled diabetes, and metastatic spread from primary infections such as liver abscess. However, invasive liver abscess syndrome caused by hvKp notably affects immunocompetent individuals, as observed in our patient. In our case, the patient was a young, immunocompetent, healthy male who developed KPM, and investigations during the hospital stay revealed a liver abscess, which could be the primary source of neurological spread. The patient was continuously monitored for neurological conditions as he developed right-sided hemiplegia and facial nerve weakness. A CT scan of the brain revealed no evidence of acute stroke. Unfortunately, MRI could not be performed owing to financial constraints. Neurological sequelae are common after bacterial meningitis and most frequently include focal neurological deficits, hearing loss, cognitive impairment, and epilepsy [8]. Our patient showed significant improvement in right-sided weakness, with power returning to almost normal, but had minimal hearing loss at the time of discharge. The evolving resistance of *K. pneumoniae* to various antibiotics challenges treating infection. According to a recent thorough analysis of carbapenem-resistant *K. pneumoniae* (CRKP) infections, the mortality rate of these infections, especially those caused by hvKp strains, is noticeably higher than that of other bacterial infections. This study indicated that the predictive markers for death from CRKP infection included length of hospitalization, C-reactive protein-to-prealbumin ratio, CRP/PA ratio, and CRKP infection status [9]. In our patient, blood and CSF cultures confirmed KPM. Genetic hypervirulence testing could not be performed due to resource constraints. The antibiotic susceptibility profile was promising, with a good response to all antibiotics; meropenem was initiated at 1 g every eight hours and subsequently increased to 2 g every eight hours. Despite an appropriately aggressive antibiotic regimen, our patient continued to be febrile, which led us to investigate this further. A contrast-enhanced CT scan of the abdomen performed on day five revealed a 6 × 7 cm hypodense lesion with peripheral enhancement in the left hepatic lobe, consistent with a liver abscess, along with a small lesion in segment IVb. Percutaneous drainage of the liver abscess was performed. The association between invasive *K. pneumoniae* liver abscess syndrome and KPM is well documented. Invasive *K. pneumoniae* liver abscess (IKLAS) is a severe infection characterized by liver abscesses caused by presumptive hypervirulent *K. pneumoniae* (hvKP) strains that frequently infect individuals without predisposing conditions [1]. These hypervirulent strains have an increased ability to manufacture virulence factors such as mucins and hyaluronic acid (via the rmpA gene), which facilitates their ability to infect immunocompetent hosts [10]. Early percutaneous drainage of the liver abscess resulted in tremendous and fast recovery, and the patient was successfully discharged from the ICU. This case has several limitations. First, specialized hypervirulent testing and the string test were not performed at our facility, and therefore the hypervirulent phenotype of the *K. pneumoniae* isolate could not be definitively established. Due to financial constraints, a brain MRI was not performed, limiting detailed radiological assessment of the neurological complication. Nevertheless, the clinical presentation, microbiological findings, and identification of hepatic abscess support the diagnosis of invasive *K. pneumoniae* infection

## Conclusions

This case highlights that community-acquired KPM can present as a severe, life-threatening infection with a substantial risk of neurological sequelae. Early diagnosis, prompt initiation of appropriate antimicrobial therapy, and thorough investigation of the underlying source, particularly liver abscesses, are essential for improving patient outcomes. Timely source control measures and appropriate antimicrobial treatment may result in substantial neurological recovery, even in patients with severe neurological involvement. 
